# Visfatin/Nampt: An Adipokine with Cardiovascular Impact

**DOI:** 10.1155/2013/946427

**Published:** 2013-06-16

**Authors:** Tania Romacho, Carlos F. Sánchez-Ferrer, Concepción Peiró

**Affiliations:** ^1^Paul-Langerhans Group of Integrative Physiology, German Diabetes Center, Auf'm Hennekamp 65, 40225 Düsseldorf, Germany; ^2^Departamento de Farmacología y Terapéutica, Facultad de Medicina, Universidad Autónoma de Madrid, Arzobispo Morcillo 4, 28029 Madrid, Spain

## Abstract

Adipose tissue is acknowledged as an endocrine organ that releases bioactive factors termed adipokines. Visfatin was initially identified as a novel adipokine with insulin-mimetic properties in mice. This adipokine was identical to two previously described molecules, namely, pre-B cell colony-enhancing factor (PBEF) and the enzyme nicotinamide phosphoribosyltransferase (Nampt). Enhanced circulating visfatin/Nampt levels have been reported in metabolic diseases, such as obesity and type 2 diabetes. Moreover, visfatin/Nampt circulating levels correlate with markers of systemic inflammation. In cardiovascular diseases, visfatin/Nampt was initially proposed as a clinical marker of atherosclerosis, endothelial dysfunction, and vascular damage, with a potential prognostic value. Nevertheless, beyond being a surrogate clinical marker, visfatin/Nampt is an active player promoting vascular inflammation, and atherosclerosis. Visfatin/Nampt effects on cytokine and chemokine secretion, macrophage survival, leukocyte recruitment by endothelial cells, vascular smooth muscle inflammation and plaque destabilization make of this adipokine an active factor in the development and progression of atherosclerosis. Further research is required to fully understand the mechanisms mediating the cellular actions of this adipokine and to better characterize the factors regulating visfatin/Nampt expression and release in all these pathologic scenarios. Only then, we will be able to conclude whether visfatin/Nampt is a therapeutical target in cardiometabolic diseases.

## 1. Introduction

The adipose tissue (AT) is no longer considered a triglyceride-storing depot but a real endocrine organ that synthesizes and secretes a wide range of diverse bioactive factors, called adipokines. These adipokines can act locally within the adipose tissue, but can also trigger an effect on distant organs or tissues through their release to the systemic circulation. Adipokines comprise cytokines and chemokines, such as tumor necrosis factor- (TNF-) *α*, interleukins (IL), or monocyte chemotactic protein-1 (MCP-) 1, vasoactive and coagulation factors, such as angiotensinogen or plasminogen activator inhibitor-1, (PAI-1), and proteins more specifically secreted by the adipose tissue, such as leptin or adiponectin. In the last years, the number of the adipokines has notably increased with novel adipokines such as visfatin/Nampt, resistin, apelin, or dipeptidyl peptidase-4 (DPP-4) among many others [1–4].

 Adipokines exhibit a wide range of functions including the regulation of food intake and body weight homeostasis, insulin sensitivity, reproduction, immunity, inflammation, or vascular homeostasis [4, 5]. In obesity and type 2 diabetes mellitus, there is an imbalanced adipokine production that has been locally associated with AT inflammation, and the onset of insulin resistance, but also with chronic systemic inflammation, cardiovascular disease, and endothelial dysfunction [6]. In the context of metabolic diseases, adipokines are not only key mediators in the complex crosstalk between AT with other insulin-sensitive organs such as liver, skeletal muscle, and AT itself, but also have an impact on the cardiovascular system. Therefore, in the last years, there is a growing interest in the potential role of adipokines as biomarkers of low-grade inflammation and metabolic-related cardiovascular complications. In this review, we will specifically focus on the adipokine visfatin/Nampt and its impact on the cardiovascular (CV) system.

## 2. Visfatin/Nampt: An Adipokine and Beyond

In 2005, Fukuhara et al. firstly described visfatin as an adipokine exhibiting insulin mimetic properties in mice [2]. However, the expectation initially arisen by the novel adipokine was soon blunted when the authors had to retract their paper due to the lack of reproducibility of the hypoglycemic properties [5]. Visfatin was found identical to pre-B cell colony-enhancing factor (PBEF), a previously described cytokine promoting maturation on early B-lineage precursor cells [6]. 

The term visfatin refers to visceral fat, since it was initially suggested that visfatin was mainly produced in visceral fat compared to subcutaneous fat in both mice and humans [2]. Nevertheless, other groups have later reported similar visfatin levels in human subcutaneous and visceral fat tissue [7, 8]. Importantly, visfatin is also found in other fat depots such as perivascular and epicardial fat [9, 10], where it might exert a paracrine cardiovascular effect as will be further discussed. Within the adipose tissue, visfatin is not only synthesized and released by adipocytes but also by inflammatory cells, like activated macrophages, whose infiltration in AT is markedly increased in relation with obesity [11].

 Visfatin displays intrinsic enzymatic activity as a nicotinamide phosphoribosyltransferase (Nampt), as reported in 2002 by Rongvaux et al. [12]. In mammals, intracellular Nampt catalyzes the rate-limiting step in the salvage pathway leading to the synthesis of nicotinamide adenine dinucleotide (NAD^+^), an essential coenzyme in multiple cellular redox reactions [13, 14]. More specifically, Nampt synthesizes nicotinamide mononucleotide (NMN) and inorganic pyrophosphate by condensing nicotinamide and 5-pyrophosphoribosyl-1-pyrophosphate (PRPP). In a second step, NMN is transformed into NAD by nicotinamide mononucleotide adenylyltransferase (Nmnat) [14].

 In mammals, two isoforms of Nampt have been described. Intracellular Nampt (iNampt), which plays a central role in maintaining the activity of NAD-dependent enzymes, is implicated in the regulation of cellular metabolism in response to nutrient availability, maturation and cell survival [14–17]. On the contrary, the other isoform, extracellular Nampt (eNampt), is secreted by different cell types [18]. In this review, we will mainly focus on eNampt as this isoform may represent a mediator of interorgan crosstalk. 

In the current literature, we find indistinctly the terms visfatin/Nampt/PBEF to refer to this adipokine. According to the HUGO Gene Nomenclature Committee (HGNC) and the Mouse Genomic Nomenclature Committee (MGNC) the official nomenclature of the gene and the protein is Nampt [19]. However, since the Nampt activity is not always mediating visfatin/Nampt effects, we will employ visfatin/Nampt throughout this review. 

 In the last years, visfatin/Nampt has indeed arisen as a multifaceted and ubiquitously expressed molecule that exerts multiple biological actions beyond the adipose tissue [20, 21]. Indeed, among adipocytes, visfatin/Nampt is expressed in a wide range of cell types from the immune system, chondrocytes, and amniotic epithelium cells among others [11, 22, 23]. In 1994, Samal et al. initially provided qualitative evidence for visfatin expression in lysates from human heart, pancreas, liver, and skeletal muscle at mRNA levels [6]. More recently, visfatin protein expression has been reported in human myoblasts [24] and human hepatocytes, from which visfatin is actively secreted [25]. Moreover, visfatin expression in liver and skeletal muscle has been further confirmed by other studies [26, 27], raising the possibility that these organs could be potential sources of visfatin plasma levels and thus contributing to metabolic interorgan crosstalk.

## 3. Circulating Visfatin/Nampt as a Biomarker of Inflammation, and CV Disease

### 3.1. Obesity, Type 2 Diabetes and the Metabolic Syndrome

Obesity and type 2 diabetes represent two independent risk factors for inflammation-related atherothrombotic diseases. In the context of metabolic diseases, most studies have reported elevated circulating levels of visfatin/Nampt in different clinical conditions, such as obesity, type 2 diabetes mellitus, and the metabolic syndrome which represent independent risk factors for inflammation-related atherothrombotic diseases [28, 29]. Nevertheless, as reviewed by others, there are conflicting results in different reports studying the variation of visfatin/Nampt levels in these disease states, where circulating visfatin/Nampt levels have been found unmodified or even lower compared to healthy controls [29, 30]. However, in the last years, several studies have established positive associations between enhanced circulating visfatin/Nampt levels and atherogenic inflammatory diseases, therefore supporting a role for visfatin as a potential biomarker of cardiovascular complications associated to metabolic disorders.

 In the last years, visfatin/Nampt has been proposed as a marker of endothelial dysfunction, an initial and crucial step in the progression of the atherosclerotic process [31]. In type 2 diabetic patients, it has been reported a strong correlation between visfatin/Nampt levels and impaired vascular endothelial function determined as brachial artery flow-mediated dilation (FMD) and creatinine clearance [32]. Although in this latter work, no association was found between inflammatory markers, such as high-sensitivity C-reactive protein (hsCRP) and fibrinogen, neither with atherosclerosis, as evaluated by assessing intima-media thickness (IMT), and so the authors concluded that visfatin/Nampt is more likely not a surrogate marker of inflammation nor atherosclerosis. Additionally, Uslu et al. have found that type 2 diabetic patients display enhanced circulating visfatin levels which positively correlate with enhanced levels of the marker of endothelial dysfunction homocystein. Surprisingly, this work could not find a positive correlation between visfatin levels in type 2 diabetic patients and asymmetric dimethylarginine (ADMA), the major endogenous inhibitor of the endothelial nitric oxide synthase (eNOS) [33]. Homocysteine has been proposed as an intermediate factor in the relationship between endothelial dysfunction and renal function [34].

In both patients with the metabolic syndrome and type 2 diabetes, it has been suggested that enhanced visfatin/Nampt levels are associated with advanced carotid atherosclerosis, estimated as the intima-media thickness (IMT) in this artery [35, 38]. Indeed, Kadoglou et al. have proposed visfatin/Nampt circulating levels as a marker of advanced carotid atherosclerosis for type 2 diabetic patients. In morbid obese patients, epicardial fat thickness as assessed by echocardiography was related to enhanced visfatin/Nampt and plasminogen activator inhibitor-1 (PAI-1) levels as well as visceral obesity [36].

 On the contrary, some authors claim that high visfatin/Nampt levels, instead of depicting changes in the atherosclerotic process are more likely reflecting changes in the systemic inflammation in patients with renal and cardiovascular disease. Thus, in patients undergoing hemodialysis, visfatin/Nampt levels are associated with high-sensitivity C-reactive protein (hsCRP), considered one of the most powerful predictors of atherosclerosis and vascular death, but not with other parameters of atherosclerosis such as ADMA levels, aortic pulse wave, brachial pressure index in the ankle, or the percentage of calcification in the abdominal aortic wall [45]. In this line, Visfatin/Nampt expression is upregulated in circulating blood monocytes from obese type 2 diabetic patients compared to nondiabetic obese patients [50], indicating that enhanced visfatin/Nampt expression may be related to type 2 diabetes rather than obesity. On the contrary, Oki et al. [51] have reported that serum visfatin/Nampt levels positively correlate with inflammatory markers, independently of the insulin resistance state. Hence, the significance of visfatin/Nampt levels variations in metabolic diseases remains to be more accurately addressed. However, it seems clear that visfatin/Nampt levels are positively associated to a series of inflammatory conditions, independently of other potential metabolic implications. Thus, serum visfatin/Nampt levels have been positively correlated with circulating inflammatory markers, such as IL-6, CRP, and MCP-1 [40, 45, 51]. 

### 3.2. Chronic Kidney Disease

Diabetes is the main risk factor for the development and progression of chronic kidney disease (CKD) [52, 53]. In patients with CKD, visfatin/Nampt levels positively correlate with soluble markers of endothelial dysfunction such as vascular, intercellular, and melanoma cells adhesion molecule-1 (VCAM-1, ICAM-1, and MCAM-1, resp.) [43, 44]. The relation between visfatin/Nampt levels and endothelial function in CKD is not merely descriptive, but may also have a functional impact, since visfatin/Nampt levels negatively correlate with endothelial function estimated as flow-mediated dilation (FMD) in brachial artery or glomerular filtration rate (GFR) [41, 42]. Indeed, the improvement of endothelial function after kidney transplantation correlates with a reduction in circulating visfatin/Nampt levels [46]. 

### 3.3. Polycystic Ovary Syndrome

Visfatin/Nampt has been recently proposed as a candidate in the pathogenesis of endothelial dysfunction in polycystic ovary syndrome (PCOS), the main androgen excess disorder in women [39]. PCOS is characterized by obesity, insulin resistance, and endothelial dysfunction. It has been previously proposed that PCOS is associated with a dysfunctional secretion pattern of adipokines. In this regard, enhanced visfatin/Nampt levels and expression in AT have been previously reported in PCOS [8, 54]. Interestingly, enhanced visfatin/Nampt levels have been associated with reduced brachial artery flow-mediated vasodilatation in patients with PCOS and significantly predicted impaired endothelial function [39]. Similar to what described for type 2 diabetic patients by Takebayashi et al. visfatin/Nampt levels did not correlate with inflammatory markers as hsCRP or carotid IMT.

### 3.4. Preeclampsia

Preeclampsia is a hypertensive disorder in pregnancy and is associated with increased cardiovascular disease (CVD) risk later in life and is a major cause of maternal and fetal mortality and morbidity in pregnancy [55]. Preeclampsia shares cardiovascular risk factors with the metabolic syndrome such as subclinical inflammation, insulin resistance, and obesity. Since visfatin/Nampt levels are enhanced in all these pathological conditions, several groups have aimed to determine if visfatin/Nampt may contribute to preeclampsia. Thus, different groups have reported enhanced maternal visfatin/Nampt serum levels in preeclamptic patients compared to their matched pregnant controls [37, 56, 57]. Furthermore, Fasshauer et al. also found positive correlations between visfatin/Nampt serum concentrations and age, blood pressure, creatinine, free fatty acids (FFA), and the inflammatory markers IL-6 and CRP [37]. On the contrary, other groups suggest that decreased expression of visfatin, as a proangiogenic factor, may be associated with the pathogenesis of preeclampsia. Thus, Hu et al. have reported that maternal plasma visfatin/Nampt levels were downregulated in women with mild preeclampsia and to a higher extent in women with severe preeclampsia [58]. In this line, it has been recently reported by Kim et al. that in placental biopsies visfatin/Nampt expression is reduced compared to the visfatin/Nampt levels in placentas from normotensive women [59]. Reduced placental perfusion is an initial event in preeclampsia, initiating a sequence of events leading to altered vascular function and hypertension. Thus, decreased placental perfusion-induced endothelial dysfunction has been suggested as the cause leading to progressive vasoconstriction, hyperresponsiveness, and impaired relaxation of uterine arcuate arteries [60]. However, it has been recently demonstrated that impaired placental perfusion is not the mechanism responsible for visfatin/Nampt induction in preeclampsia [56]. Ognjanovic et al. have demonstrated that visfatin/Nampt is also locally expressed in foetal membranes and secreted from amniotic epithelium cells in the human placenta and that its secretion is increased in response to proinflammatory stimuli such as lipopolysaccharide (LPS) and IL-1*β* [23]. On the other hand, exogenous administration of visfatin/Nampt to human foetal membranes leads to an increase in inflammatory cytokines such as IL-1*β*, TNF-*α*, and IL-6 [23, 61]. Visfatin/Nampt also prevented actinomycin D-induced apoptosis, therefore suggesting a protective role for visfatin/Nampt in preventing apoptosis induced by chronic distension, labor, or infection in the placenta [61]. Thus, whether enhanced visfatin/Nampt levels is a deleterious factor promoting endothelial dysfunction in placenta and leading to preeclampsia or, if on the contrary, is a beneficial factor preventing apoptosis under inflammatory conditions and promoting angiogenesis in the placenta, or if it is just a biomarker, is still an open question [62].

### 3.5. Acute Coronary Syndromes

Enhanced circulating visfatin/Nampt levels have been proposed to correlate with the development of atherosclerotic plaques, and thus visfatin/Nampt has been proposed as a marker of atherosclerosis by several groups [35, 40]. It has been described that in coronary artery diseases (CADs), and more specifically in acute coronary syndrome, circulating inflammatory markers such as IL-6 and MCP-1 positively correlate with visfatin/Nampt levels [40]. In patients with coronary artery disease (CAD) and acute myocardial infarction, a positive association between visfatin/Nampt expression and unstable atherosclerotic lesions has been established [63]. Interestingly, another positive correlation has been established between visfatin/Nampt expression in both pericardiac and periaortic fat and coronary artery atherosclerosis [64], which underpins that not only circulating but also perivascular visfatin/Nampt may exert an important paracrine effect promoting the development of atherosclerotic lesions. A higher expression of visfatin/Nampt has been found not only in the smooth muscle within atherosclerotic plaques [64] but also in foam cells of unstable plaques from patients that suffered an acute myocardial infarction [63]. Therefore, it has been proposed that visfatin/Nampt localization within the lesions may be related to atherosclerotic plaque destabilization [63]. In this line, Yu et al. have recently described that visfatin/Nampt levels are upregulated in the circulation of patients suffering a ST-segment elevation myocardial infarction (STEMI), the most acute form of MI. Furthermore, the authors found enhanced visfatin/Nampt expression in macrophages present in the coronary rupture plaques. These results support the hypothesis that leukocytes-derived visfatin/Nampt may play a role in the pathogenesis of coronary plaques rupture. Importantly, this group has previously proposed that enhanced circulating visfatin/Nampt levels associate with the occlusion of infarct-related artery (IRA) and circulating hsCRP levels [48] and associate with the degree of myocardial damage [49]. In the light of the bulk of evidence presented herein, visfatin/Nampt arises as a relevant molecule promoting plaque destabilization and rupture in different types of acute coronary syndromes.

### 3.6. Cerebrovascular Diseases

Visfatin/Nampt has also been proposed to play a role in cerebrovascular diseases [65]. Thus, Lu et al. have demonstrated that plasma visfatin/Nampt was increased in a Chinese population of patients with ischemic stroke and correlated with hsCRP levels in these patients [47]. In this line, it has been recently proposed that aging decreases intracellular visfatin/Nampt expression in the murine brain, in parallel to increased visfatin/Nampt circulating plasma levels, and may contribute to endothelial dysfunction in the brain [66]. 

### 3.7. Non-metabolic Chronic Inflammatory Diseases

In the context of other non-metabolic chronic inflammatory diseases characterized by systemic inflammation enhanced visfatin/Nampt levels have been additionally reported. Thus, visfatin/Nampt circulating levels are enhanced in osteoarthritis [67, 68], Crohn's disease, and ulcerative colitis [20, 69]. Moreover, in patients with acute lung injury (ALI), visfatin/Nampt is currently considered a biomarker of this disease [70]. Visfatin/Nampt also seems to play a role in several types of infections like sepsis [71] or intrauterine infection (chorioamnionitis) [61, 72]. Additionally, visfatin/Nampt also may have a crucial role in autoimmune inflammatory diseases since enhanced visfatin/Nampt levels have been reported in psoriasis [53], rheumatoid arthritis (RA) [43], and inflammatory bowel disease (IBD). Interestingly, visfatin/Nampt has been identified as a novel “universal marker of chronic inflammation” whose RNA is upregulated in the mononuclear cells from peripheral blood from patients suffering any of these three chronic inflammatory diseases, which allows to discriminate patients with chronic inflammation and healthy controls mRNA expression levels [73]. 

In RA, it has been proposed that visfatin/Nampt can be a potent mediator of inflammation [67]. However, Senolt et al. provided evidence for a positive correlation between the levels of serum visfatin/Nampt and total number of B cells in RA, more than activity of the disease [74]. Indeed both visfatin/Nampt levels and B cell number were reduced after treatment with rituximab. 

 In patients with psoriasis, it has been speculated that visfatin enhanced levels may lead to atherosclerosis and vascular complications as frequent comorbidities found in this disease [75]. Analogously, it has been suggested that the proinflammatory and matrix-degrading activities of visfatin reported in the context of RA [67] may contribute to the enhanced risk for CVD in these patients [76]. However, the relation between enhanced visfatin levels and CVD in these inflammatory diseases needs to be further explored.

**Figure 1:**
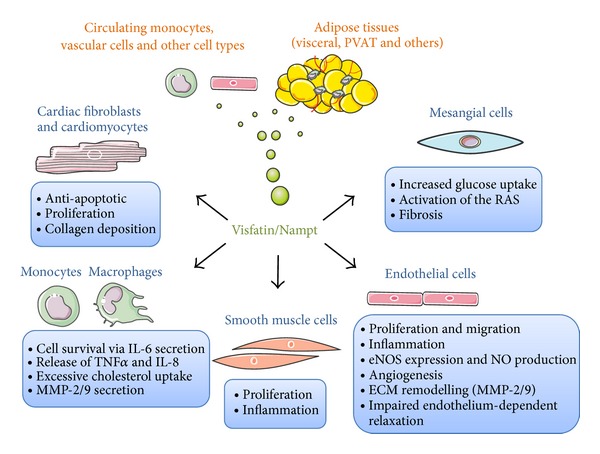
Diagram summarizing the main reported direct actions of visfatin/Nampt in cells in the cardiovascular system, namely, cardiac fibroblast and cardiomyocytes, mesangial cells, monocytes and macrophages, smooth muscle cells, and endothelial cells. eNOS: endothelial nitric oxide synthase, IL-8: interleukin-8, MMP-2/9: matrix metalloproteinase-2/9, NO: nitric oxide, RAS: renin-angiotensin system, and TNF-*α*: tumor necrosis factor-*α*.

In the light of all these studies, we can conclude that visfatin/Nampt is upregulated and may play a role in both atherosclerosis and endothelial dysfunction (Table 1). But beyond being a marker for CVD, growing evidence supports a role for visfatin/Nampt as direct factor triggering vascular injury. Here, we will try to summarize the most relevant previous and recent evidences about visfatin/Nampt direct actions on the cardiovascular system (Figure 1). More specifically, we will focus on visfatin/Nampt effect on proliferation, and angiogenesis of vascular cells, visfatin/Nampt inflammatory effects, extracellular matrix degradation and finally apoptosis/cell survival.

## 4. Direct Cardiovascular Actions of Visfatin/Nampt

### 4.1. Cell Proliferation and Angiogenesis

Proliferation of vascular smooth muscle cell (VSMC) is a hallmark of the development of atherosclerotic lesions. Importantly, perivascular visfatin/Nampt can act as a growth factor in vascular smooth muscle cells, promoting cell proliferation in rat aortic smooth muscle cells though Nampt enzymatic activity [10]. Visfatin/Nampt emerges as a player in the development and progression of atherosclerotic lesions by directly promoting smooth muscle cell proliferation. 

 Aberrant angiogenesis is now considered a feature of the atherogenic process in both coronary and carotid diseases [77]. In this line, it has also been described that visfatin/Nampt can promote endothelial cell proliferation, migration, and capillary tube formation in a concentration-dependent manner in HUVEC [78–81]. These proliferative effects of visfatin/Nampt seem to be mediated, or at least partially mediated, by a master molecule in endothelial proliferation and neovessel formation: vascular endothelial cell growth factor (VEGF) [78]. Thus, visfatin/Nampt upregulates VEGF synthesis and secretion as well as the expression of the VEGF receptor 2, which has been proposed to mediate the angiogenic actions of VEGF [78, 82]. Besides VEGF, visfatin/Nampt upregulates the production of other pro-angiogenic soluble factors, such as fibroblast growth factor-2 (FGF-2), MCP-1, and IL-6, in endothelial cells [82–84]. Indeed, both MCP-1 and FGF-2 have also been identified as mediators of visfatin/Nampt-induced angiogenesis [82, 83]. Beyond *in vitro* studies, the angiogenic activities of visfatin/Nampt have been demonstrated in *ex vivo* and *in vivo *approaches [78, 79]. Thus, visfatin/Nampt is able to induce the formation of functional neovessels in chick chorioallantoic membrane and mouse Matrigel plug [79]. In this line, it has been described that the injection delivery of a plasmid containing visfatin/Nampt improved limb perfusion in a mouse undergoing unilateral hindlimb ischemia [80]. 

 Furthermore, visfatin/Nampt enhances the expression, protein levels and activity of matrix metalloproteinases, MMP-2/9, which are enzymes promoting angiogenesis through the degradation of the extracellular matrix, while it decreases the levels of their tissue inhibitors, TIMP-1 and -2, respectively [78]. The impact of visfatin on matrix remodelling will be discussed in more detail below.

 The forementioned proangiogenic properties of visfatin/Nampt make of this adipokine a potential therapeutic candidate in diseases where neovascularisation is necessary to overcome restricted blood flow supply such as ischemic stroke or macrovascular peripheral limb ischemia. Very recently, Kim et al. have proposed that visfatin/Nampt proangiogenic effects are mediated by the induction of thromboxane synthase (TSA) with the subsequent TXA_2_ release by endothelial cells (HMECs and HUVECs) [85]. The authors more specifically demonstrate that visfatin/Nampt upregulation of IL-8 via TXA_2_ is the responsible mechanism for visfatin/Nampt-induced angiogenesis [85].

 It has been recently proposed that visfatin/Nampt exerts a neuroprotective role for photothrombosis-induced ischemia with both *in vitro* and *in vivo* approaches/models [86]. Thus, heterozygous visfatin/Nampt knockout mice (Pbef^+/−^) display larger size of ischemic lesions than wild-type mice [86]. On the other hand, visfatin/Nampt may contribute to exacerbated angiogenesis leading to ischemic heart disease, diabetes, or atherosclerosis and may therefore arise as a novel pharmacological target for treating such conditions. 

 Visfatin/Nampt proangiogenic actions also promote tumor growth. Thus, circulating visfatin/Nampt levels are enhanced in several malignancies such as endometrial [87], gastric, or colorectal cancers [88, 89]. Inhibition of NAD enzymatic activity is an emerging therapeutic strategy for cancer treatment [25, 90]. In this context, two Nampt inhibitors, APO866 (or FK866) and CSH-828, are being used in clinical trials as NAD-depleting anticancer agents [91, 92]. 

 Visfatin/Nampt proliferative effects are not restricted to the vascular wall, since visfatin/Nampt also promotes proliferation in rat cardiac fibroblasts. The proliferation of cardiac fibroblasts together with an excessive accumulation of extracellular matrix represents the basis of myocardial fibrosis. *In vitro* stimulation of cardiac fibroblasts by visfatin/Nampt requires the activation of Akt/PKB and the MAPKs p38 and JNK, but not ERK 1/2 [93]. Pillai et al. have described that cardiac-specific overexpressing Nampt transgenic mice show increased cardiac fibrosis. Moreover, both recombinant and adenoviral Nampt delivery increased proliferation *in vitro* in rat cardiomyocytes, which was inhibited by Nampt-blocking antibody [94]. Taking into account that visfatin/Nampt is expressed in periadventitial and apical epicardial adipose tissues [64], and with the recent evidence that it is also secreted by rat cardiomyocytes [94], not only circulating visfatin/Nampt but also visfatin/Nampt locally produced in the CV system could play a detrimental role in promoting myocardial fibrosis and remodeling.

### 4.2. Inflammation

Growing scientific evidence supports that visfatin/Nampt can directly promote vascular inflammation by activating different cell types including endothelial cells and vascular smooth muscle cells. Moreover, visfatin/Nampt can also contribute to vascular inflammation through its immunomodulatory properties on immune cells [69]. Thus, visfatin/Nampt can exert direct actions on monocytes. Hence, visfatin/Nampt promotes the synthesis and release of pro-inflammatory cytokines, such as tumor necrosis factor- (TNF-) *α* and IL-8, by peripheral mononuclear cells [63]. Additionally, visfatin/Nampt promotes macrophage survival [95], which may help perpetuating vascular inflammation.

 In cultured human vascular smooth muscle cells, our group demonstrated for the first time that visfatin/Nampt could directly exert inflammatory effects. Thus, exogenous administration of visfatin/Nampt activates ERK 1/2 and NF-*κ*B, resulting in enhanced expression of the inducible nitric oxide synthase (iNOS) [96]. iNOS is a pro-inflammatory enzyme contributing to dysregulated NO production and subsequent peroxynitrite formation. Thus, iNOS induction plays a key role in endothelial dysfunction and vascular injury in diabetes-related vascular complications [104]. 

Several reports suggest that visfatin/Nampt can additionally promote endothelial activation. In HUVEC, visfatin/Nampt activates the inflammation-related transcription factor NF-*κ*B [82, 84, 97] and promotes the expression of cell adhesion molecules, such as ICAM-1, VCAM-1, or E-selectin [84, 97], as key molecules implicated in leukocyte recruitment and early proatherosclerotic events [105]. Visfatin/Nampt further upregulates the release of several cytokines and chemokines by endothelial cells, including IL-6, IL-8, or MCP-1 and its putative receptor CCR2 [84, 98], and thus promotes the adhesion of human THP-1 monocytes to endothelial cells [98]. The MAPK ERK 1/2 and p38, as well as PI3K and the intracellular generation of reactive oxygen species, have been proposed as responsible molecules in endothelial cell inflammation induced by visfatin/Nampt [97, 98].

Nicotinamide adenine dinucleotide phosphate (NADPH) oxidase is a superoxide anion-generating and proinflammatory enzyme closely associated to endothelial dysfunction [106]. Boini et al. have proposed that visfatin/Nampt induces the activation of NADPH oxidase, increasing superoxide anion production resulting in the disruption of microtubular networks in GECs and increased glomerular permeability [101]. Analogously, visfatin/Nampt triggers NADPH oxidase subunits assembly and activation through lipid rafts in bovine coronary artery endothelial cells [107], thereby contributing to endothelial dysfunction in coronary circulation. Alternative to NADPH activation, it has been suggested that visfatin/Nampt deleterious effect on diabetic nephropathy may be due in part to the activation of intrarenal renin-angiotensin system [108]. Inappropriate activation of the renin-angiotensin system is known to be implicated in CV diseases related to CKD. Thus, it has been reported that visfatin/Nampt leads to activation of the renin-angiotensin system by upregulating the expression levels of renin, angiotensinogen and angiotensin I and II in a dose-dependent manner in cultured rat mesangial cells [108].

 In another clinical condition such as myocardial infarction, and more specifically in STEMI, visfatin/Nampt circulating levels and intracellular expression in macrophages and monocytes are enhanced [109]. Thus, Chiu et al. conclude that male patients with STEMI show increased visfatin/Nampt expression in leukocytes, which may aggravate the development of instability of atherosclerotic plaques [109]. Interestingly, Zhou et al. have recently reported that visfatin/Nampt promotes lipid accumulation mainly through excessive cholesterol uptake in RAW264.7 macrophages and in peritoneal macrophages isolated from ApoE knockout mice and accelerates the process of atherosclerosis mainly through modulating the expression of the macrophage scavenger receptor class A (SR-A) and CD36 [103] (Table 2).

### 4.3. Extracellular Matrix

Adya et al. reported that on one hand visfatin/Nampt upregulated the expression and activity of the matrix metalloproteinases (MMP)-2/9 and downregulated the expression of the inhibitors of these MMPs such as TIMP-1 and -2, in monocytes and endothelial cells [78]. MMP-2 and -9 are pivotal enzymes in the degradation of extracellular matrix (ECM), thus facilitating atherosclerotic plaque vulnerability [110].

Cardiac fibrosis is the consequence of excessive accumulation of ECM. As we have previously mentioned, visfatin/Nampt may also contribute to cardiac fibrosis. Thus, visfatin/Nampt not only promotes proliferation in rat cardiac fibroblasts but also upregulates the mRNA expression and protein levels of procollagen I and II in this cell type, leading to enhanced types I and III collagen release [93]. 

Visfatin/Nampt has also been proposed to promote renal fibrosis, a typical feature of CKD. In the context of CKD, visfatin/Nampt promoted fibrosis in rat mesangial cells by upregulating the synthesis of key profibrotic molecules such as transforming growth factor-*β*1 (TGF-*β*1), PAI-1 and type I collagen, thus increasing the risk for CVD [111]. These results provide evidence for visfatin/Nampt as a novel factor promoting cardiac and renal fibrosis whether directly upregulating procollagen and MMPs secretion or indirectly contributing to fibrosis by upregulating the secretion of other classic fibrosis mediators such as TGF-*β* or PAI-1.

### 4.4. Cell Survival/Apoptosis

There are conflicting results regarding the role of visfatin/Nampt in the regulation of cell survival and apoptosis. On one hand, intracellular visfatin/Nampt plays a central role in maintaining the activity of NAD-dependent enzymes regulating cellular metabolism [14–17]. On the other hand, extracellular visfatin/Nampt exerts antiapoptotic effects and promotes cell survival in several cardiovascular cells. Thus, it has been demonstrated that visfatin/Nampt attenuates cell apoptosis induced by hydrogen peroxide in human endothelial cells [99], rat VSMC [10], and in both rat cardiac fibroblasts [93] and cardiomyocytes [94]. 

Several works have suggested that visfatin/Nampt could exert direct cardioprotective effects. In cultured murine cardiomyocytes undergoing hypoxia and reoxygenation, visfatin/Nampt administered at the time of reoxygenation, triggered delayed cell death, due at least in part to a delayed opening of the mitochondrial permeability transition pore (mPTP) by oxidative stress. The mPTP is a nonspecific mitochondrial channel, whose opening in the first minutes of reperfusion is a critical determinant of cardiomyocyte death [112]. Cardiac specific Nampt overexpression in mice has been proposed to prevent myocardial injury in response to myocardial ischemia and reperfusion [113]. Intravenous administration of visfatin/Nampt at the time of reperfusion reduced the infarct size in a mouse model of ischemia-reperfusion [114]. On the contrary, Pillai et al. have reported that both exogenous visfatin/Nampt and visfatin/Nampt overexpressions promote cardiac hypertrophy and adverse ventricular remodeling [94].

 On the other hand, intracellular Nampt activity is acknowledged to play a central role in cell maturation and survival in smooth muscle cells *in vitro* [16, 17]. Van der Veer et al. have demonstrated that iNampt is crucial in order to induce maturation and prevent premature senescence in human VSMC [16, 17]. 

 Zhang et al. have reported that visfatin/Nampt knockout heterozygous mice (Pbef^+/−^) subjected to photothrombosis, a cerebral ischemia model, displayed more severe brain damage and neuronal degeneration compared to wild-type mice [86]. In a model for cerebral ischemia in rats, Nampt overexpression in brain prevented neuronal death [115]. Therefore, visfatin/Nampt may represent an interesting pharmacological tool to treat both cardiac and cerebral damage derived from ischemia-reperfusion.

Importantly, visfatin/Nampt promotes macrophage survival through a mechanism involving the release of IL-6 [95]. Moreover, visfatin/Nampt also inhibits neutrophile apoptosis in clinical and experimental sepsis [71]. Thus, visfatin/Nampt can help to perpetuating cell-mediated inflammation at the sites of elevated concentrations of this adipocytokine. 

### 4.5. Vascular Tone

Impairment of endothelial vasodilatory responses is one of the earliest markers of vascular disease [116]. To date, there are only scarce and conflicting reports analyzing the direct actions of visfatin/Nampt vascular tone regulation. 

In human umbilical vein and coronary artery endothelial cells (HUVEC; HCAEC), extracellular visfatin/Nampt can enhance endothelial nitric oxide synthase (eNOS) expression and activity resulting in enhanced NO production [80]. This *in vitro* study does not support a role for visfatin/Nampt as a biomarker of endothelial dysfunction in clinical conditions such as CKD or type 2 diabetes as previously summarized. Hence, this effect on eNOS and NO production was not considered beneficial by the authors, who associated them to endothelial cell proliferation and angiogenesis considered as two deleterious proatherosclerotic events [80]. Analogously, it has been reported that visfatin, addition to an organ bath triggers the relaxation of noradrenaline (NA) precontracted rat aortic rings [117].

On the contrary, Xia et al. have shown that visfatin/Nampt inhibits the vasorelaxant response to BK in bovine coronary arteries through NADPH oxidase activation by membrane raft clustering [102]. In this line, our group has provided the first evidence for visfatin/Nampt as an active player in endothelial dysfunction in humans. Thus, we propose that besides a novel biomarker in this clinical condition, visfatin/Nampt is an active agent promoting endothelial dysfunction. We have demonstrated that visfatin/Nampt impairs endothelium-dependent relaxations in both rat and human mesenteric microvessels [100]. Importantly, the impairment of endothelium-dependent relaxation exerted by visfatin/Nampt was mediated by NADPH oxidase activation and required Nampt enzymatic activity. Visfatin/Nampt directly enhanced NADPH oxidase activity as determined in HUVEC and rat microvessels. On the contrary, both the Nampt inhibitors, APO866 or the NADPH inhibitor apocynin, prevented visfatin/Nampt-induced impaired endothelial vasorelaxation [100]. In opposition to the relaxation exerted by visfatin/Nampt in noradrenaline precontracted aortic rings described by Yamawaki et al. we did not observe any influence of visfatin/Nampt on NA-induced vasoconstriction in rat mesenteric microvessels [117]. Our results are in line with the activation of NADPH oxidase by visfatin/Nampt in bovine coronary artery endothelial cells reported by Xia et al. Therefore, more in-depth studies using both *in vivo* and *in vitro* approaches are needed to fully understand the capacity of visfatin/Nampt to impair vasodilation.

## 5. Mechanisms of Action of Visfatin/Nampt in the CV System

### 5.1. The Insulin Receptor

Visfatin/Nampt was initially proposed by Fukuhara et al. as a potential beneficial tool able to bind and activate the insulin receptor triggering glucose-lowering properties [2]. However, two years later, the authors retracted their paper, and the role of insulin receptor in visfatin/Nampt-mediated actions is still a matter of controversy [5]. In this line, the glucose-lowering actions of visfatin/Nampt have been hardly reproduced. However, in the context of CV diseases it has been proposed that the insulin receptor mediates some of the *in vitro* effects reported for visfatin/Nampt on IL-8 and TNF-*α* secretion of by human peripheral blood mononuclear cells [63], the upregulation of MMP-9 in THP-1 monocytes [63], or enhanced glucose uptake by rat mesangial cells [118]. On the contrary, a role for insulin receptor has been discarded in a wide range of cell types, including vascular cells and macrophages [10, 95, 97]. 

### 5.2. Nampt Activity

Nampt enzymatic activity has been proposed as an alternative mechanism for visfatin/Nampt actions in the CV system. Thus, the pharmacological inhibition of iNampt by APO866 decreased cell survival and promoted senescence in VSMC [17]. On the contrary, eNampt activity has been proposed to exert proatherogenic effects in smooth muscle cells. Wang et al. have reported that visfatin/Nampt from PVAT can promote smooth muscle cells proliferation through an eNampt-dependent mechanism [10]. 

 Moreover, blockade of the insulin receptor did not affect the reported inflammatory effect of visfatin/Nampt in smooth muscle cells while APO866, the inhibitor of Nampt, completely prevented visfatin/Nampt-induced inflammatory signaling [96]. Additionally, we demonstrated that visfatin/Nampt impaired endothelium-dependent relaxation in rat and human mesenteric microvessels through its intrinsic eNampt activity [100]. Thus, in the presence of the Nampt inhibitor APO866, visfatin/Nampt deleterious effects on the endothelium were prevented. This proinflammatory profile of eNampt activity is further supported by the observation that inflammatory cytokine secretion by leukocytes is regulated by eNampt activity [119]. 

 On the other hand, it has been proposed that eNampt may enhance intracellular NAD^+^ levels conferring a higher resistance of cardiomyocytes to oxidative stress in ischemia-reperfusion [113]. On the contrary, a deleterious role has also been proposed for eNampt activity in myocardial infarction. Thus, Montecucco et al. have very recently shown that treatment with of the Nampt inhibitor APO866 reduced myocardial infarct size, neutrophil infiltration, and reactive oxygen species (ROS) generation within infarcted hearts *in vivo* in a mouse model of ischemia and reperfusion [120]. Moreover, exogenous administration of APO866 prevented CXCL2-induced neutrophil recruitment and thereby reduced neutrophil-mediated tissue injury in mice *in vitro *[120]. Thus, the pharmacological inhibition of Nampt emerges as an effective therapeutic tool to reduce smooth muscle cell proliferation, inflammation, endothelial dysfunction, and oxidative stress-mediated tissue damage in myocardial infarction.

 In this line, some of the vascular and renal actions of visfatin/Nampt have also been attributed to the product of Nampt activity, NMN [10, 96, 100, 115]. NMN, the product of Nampt, exerted growth-factor-like activity in rat smooth muscle cells, through the activation of MAPKs ERK 1/2 and p38 [10]. Analogously, our group demonstrated that NMN, the product of Nampt is able to reproduce the activation of the ERK 1/2-NF-*κ*B signaling pathway induced by visfatin/Nampt, leading to iNOS upregulation in human VSMC [96]. Moreover, NMN was able to both impair endothelium-dependent relaxation in rat and human microvessels. Analogously, NMN upregulated NADPH oxidase activity in rat microvessels and HUVEC mimicking the effects exerted by visfatin/Nampt. However, NMN does not always reproduce visfatin/Nampt actions [95], indicating that visfatin/Nampt may have other additional mechanisms of action. 

 Interestingly, it has been recently proposed that visfatin/Nampt can induce prostaglandin E(2) (PGE(2)) synthesis in chondrocytes by both Nampt activity and insulin receptor activation [121]. Whether this dual mechanism of action can be extrapolated to some of the detrimental effects of visfatin/Nampt in the CV system needs to be further explored. 

### 5.3. Other Mechanisms

As stated before, PBEF was first identified as an immunomodulatory cytokine able to synergize with interleukin 7 (IL-7) and stem cell factor (SCF) to promote pre-B cell colony formation [6]. A role for insulin receptor in visfatin/Nampt-mediated cytokine release has been discarded [69]. Analogously, the upregulation of the CAMs induced by visfatin/Nampt human endothelial cells did not depend on insulin receptor activation [84, 97]. In this regard, Li et al. reported that visfatin/Nampt-induced survival of macrophages under endoplasmic reticulum stress was not mediated by Nampt activity nor through the insulin receptor [95]. Therefore, Li and other authors have proposed that the effects of visfatin/Nampt on monocytes and endothelial cells activation may be mediated by a yet unidentified receptor. Interestingly, Xia et al. have proposed that visfatin/Nampt may act as a ligand of inflammatory or death receptor [102]. Thus, they propose that visfatin/Nampt effects on the impairment of endothelium-dependent vasodilation in bovine coronary arteries may be mediated through a death receptor, which leads to the formation of the signalling platforms called membrane rafts (MRs) [102]. This opinion is supported by the observation that visfatin/Nampt enhanced acid sphingomyelinase (ASMase) activity. ASMase is an enzyme that promotes the clustering of membrane rafts (MR) resulting in MR-associated transmembrane signaling. This ASMase is translocated onto the plasma membrane via membrane proximal lysosome trafficking and fusion upon stimulation of death receptors [122]. Visfatin/Nampt pharmacological or genetic silencing prevented MR clustering and consequent formation of the MR signalling platforms or signalosomes in coronary arterial ECs. Both the pharmacological inhibition of ASMase with amitriptyline and genetic silencing with siRNA almost completely abolished visfatin/Nampt effects on endothelial injury. Moreover, the authors claim that the death receptor agonist FasL mimicked the effect exerted by visfatin/Nampt on endothelium-dependent vasodilation [102]. However, this hypothesis does not match with the proposed antiapoptotic effects of visfatin/Nampt in cardiomyocytes. Furthermore, it has been recently reported that visfatin/Nampt has a protective effect on oxygen peroxide-induced myocardial apoptosis, not by inhibiting the death receptor-dependent apoptotic pathways, but most likely acting on p53-mediated, mitochondria-dependent apoptotic signaling and via involvement of the AMPK signaling pathway in H9c2 cardiomyocytes [123].

## 6. Sources of Visfatin/Nampt with Potential CV Impact

Visceral adipose tissue was initially proposed as the main source of circulating visfatin/Nampt in humans [2]. Visfatin/Nampt plasma levels were proposed to correlate with the amount of visceral fat in humans as determined by computerized tomography [2]. Nevertheless, further evidence has demonstrated that visfatin/Nampt is expressed in similar levels in human subcutaneous AT [7, 8]. As previously mentioned, visfatin/Nampt is found in perivascular and epicardial fat and can therefore act in a paracrine manner on the CV system [9, 10]. Indeed, epicardial AT explants virtually release the same levels as upper abdominal SAT [9]. Epicardial fat (EF) is now considered a true kind of visceral adipose tissue (VAT) depot surrounding the heart. The impact of epicardial fat remains relatively unstudied and is now under intensive investigation in order to explore its contribution to CVD due to its ability to synthesize and release several inflammatory adipokines. Indeed, epicardial fat has been proposed as a marker of visceral adiposity [124] and as an indicator of cardiovascular risk [125]. 

 As we have previously mentioned, macrophages represent a major source of visfatin/Nampt in AT [11]. In line with the proposed inflammatory properties of visfatin/Nampt, other circulating cytokine-producing cell types have been described as sources of visfatin/Nampt, namely, activated lymphocytes, monocytes, or neutrophiles [6, 63, 71]. 

Therefore, it is not striking that visfatin/Nampt expression has been reported in macrophages from atherosclerotic plaques [35, 63, 109]. However, besides being expressed in macrophages and foam cells, visfatin/Nampt is also found in different cell types present in the vascular wall. Thus, visfatin expression has been reported in both coronary and aortic VSMCs [64]. A single report by Lovren et al. has proposed the human endothelial cells (HUVECs and HCAECs) as an additional source for visfatin/Nampt within the vascular wall [80]. In this line, visfatin mRNA expression has been reported in endothelial cells [126].

These observations suggest that, besides cells of circulating origin, other cell types from the atherosclerotic lesion can represent an additional source of this adipocytokine, which may then reach high local concentrations within the vascular wall. 

 Cardiomyocytes have very recently been discovered as a visfatin/Nampt-secreting cell type [94]. Indeed, visfatin/Nampt secretion by cardiomyocytes is enhanced in response to stress and has been proposed to induce cardiac hypertrophy and fibrosis through the activation of JNK1, p38, and ERK. These results suggest that visfatin/Nampt is a positive regulator of cardiac hypertrophy and adverse ventricular remodeling [94]. Therefore, although VAT may be the main source contributing to circulating levels of visfatin/Nampt, we cannot ignore the impact of visfatin/Nampt from other adipose depots such as PVAT or EF that can act in a paracrine manner in the CV system. Moreover, visfatin/Nampt from activated immune cells, prone to be recruited into the vascular wall, or locally synthesized visfatin/Nampt by cardiomyocytes or vascular cells may also contribute in an autocrine manner to the reported impact of visfatin/Nampt in the CV system.

## 7. Conclusions

In 2005, visfatin/Nampt was identified as a novel adipocytokine with suggested beneficial effects in the context of metabolic disorders, as an insulin-mimetic with glucose-lowering properties. The retraction of Fukuhara and colleagues combined with growing evidence supporting a role for visfatin/Nampt in metabolic diseases suddenly shifted the role of this adipokine from friend to foe. However, in light of the recent advances in the field, to the question “visfatin/Nampt: friend or foe?”, the answer should be “depending on the scenario.”

As discussed in this review, there is growing clinical evidence supporting a role for visfatin as a biomarker or even a predictor of inflammation, and endothelial injury in several metabolic diseases. 

 Importantly*, in vitro* and *ex vivo* approaches have now provided evidence that visfatin/Nampt may exert direct deleterious actions on the cardiovascular system, including cell proliferation, monocyte/macrophage activation and recruitment, vascular inflammation and remodeling, all of which leading to the development of atherosclerotic lesions. In this context, the pharmacological inhibition of the cardiovascular actions of visfatin might represent a novel therapeutic approach to prevent and treat cardiometabolic complications. In the same line, the inhibition of visfatin proangiogenic actions might also be useful in treating but pathologies implying excessive neovascularization. Indeed, there are currently ongoing clinical trials at an early phase in this direction. In this context, the pharmacological inhibition of the cardiovascular actions on the visfatin/Nampt might represent a novel therapeutic approach to prevent and treat cardiometabolic complications.

 On the other hand, beneficial actions have also been proposed for visfatin/Nampt, since the administration of visfatin/Nampt has shown beneficial effects in ischemia-related clinical conditions, such as stroke, peripheral limb ischemia, or myocardial ischemia-reperfusion, where it may become a beneficial pharmacological tool. 

Although visfatin has emerged in the last years as a promising pharmacological target in the context of cardiovascular complications, further research is still required to understand the impact of visfatin in different scenarios and clinical conditions and to evaluate the real value of visfatin as a therapeutical target in the cardiovascular system.

## Figures and Tables

**Table 1:** Summary of the main correlations reported between visfatin circulating levels and CV disease in human subjects.

Clinical condition	Main correlations found	References
↔TD2	+Endothelial dysfunction (FMD)	[32]
↑TD2	+Homocystein, ±ADMA	[33]
↑TD2	+Carotid IMT	[35]
↑Morbid obesity	+Epicardial fat thickness, +PAI-1	[36]
↑Preeclampsia	+CRP, +IL-6, +blood pressure, and +free fatty acids	[37]
↑Metabolic syndrome	+IL-6, +MCP-1, and +IMT	[38]
↑Metabolic syndrome	+Blood pressure	[29]
↑PCOS	+Endothelial dysfunction (FMD), ±hsCRP, and ±IMT	[39]
↑CAD	+MCP-1, +IL-6	[40]
↑CKD	−GFR, +TD2, and +endothelial dysfunction (FMD)	[41]
↑CKD	+GFR, +IL-6, +CRP, and +sVCAM-1	[42]
↑CKD	+sVCAM-1, +sICAM-1, and +MCAM	[43, 44]
↑Hemodialysis	±Atherosclerosis, +hsCRP	[45]
↑Renal transplantation	+Endothelial function (FMD)	[46]
↑Ischemic stroke	+hsCRP, −LDLc	[47]
↑STEMI	+Occlusion IRA, +hsCRP, and +myocardial damage	[48, 49]

↔: no change in circulating visfatin levels. ↑: enhanced circulating visfatin levels. ↓: reduced circulating visfatin levels. +: positive correlation reported, −: negative correlation reported, ±: no significant correlation reported. CAD: coronary artery disease, FMD: flow-mediated dilation, GFR: glomerular filtration rate, hsCRP: high-sensitivity C-reactive protein, IL: interleukin, IMT: intima-media thickness, IRA: infarct-related artery, LDLc: low-density lipoprotein-associated cholesterol, MCAM: melanoma cell adhesion molecule, MCP-1: monocyte chemotactic protein-1, PAI-1: plasminogen activator inhibitor-1, PCOS: polycystic ovary syndrome, sICAM: soluble intercellular adhesion molecule, sVCAM: soluble vascular cell adhesion molecule, and TD2: type 2 diabetes mellitus.

**Table 2:** Direct vascular proinflammatory actions of visfatin and their proposed underlying mechanisms.

Target cell type	Cellular actions	Mechanism of action	References
Smooth muscle cells	ERK 1/2-NF-*κ*B activation iNOS induction	Nampt activity, insulin receptor independent	[96]

Endothelial cells	NF-*κ*B activation	Insulin receptor independent	[82, 84, 97]
	IL-6, IL-8 release	N.D.	[84, 98]
	MCP-1 release	N.D.	[82, 98]
	CCR2 expression	N.D.	[82]
	ICAM-1, VCAM-1, and E-selectin	Insulin receptor independent	[84, 97]
	induction		
	MMP-2 and MMP-9 activation	N.D.	[99]
	NADPH oxidase activation	Nampt activity (HUVEC)	[100]
		Lipid rafts (BCAEC and GEC)	[101, 102]

Monocytes	Binding to endothelial cells	N.D.	[84]
	MMP-9 activation	Insulin receptor independent	[63]

Macrophages	Cell survival	STAT3/IL-6 release Nampt- and insulin receptor-independent	[95]
	Lipid accumulation	SR-A, CD36 activation	[103]

Peripheral blood mononuclear cells	Cytokine release (IL-8, TNF-*α*)	Insulin receptor	[63]

N.D.: not determined. BCAEC: bovine coronary artery endothelial cells, CCR2: chemokine receptor type 2, CD36: cluster of differentiation 36, ERK 1/2: extracellular signal-regulated kinase 1/2, GEC: glomerular endotelial cells, HUVEC: human umbilical vein endothelial cells, ICAM-1: intercellular adhesion molecule-1, IL: interleukin, MCP-1: monocyte chemotactic protein-1, MMP: matrix metalloproteinase, NF-*κ*B: nuclear factor-*κ*B, iNOS: inducible nitric oxide synthase, SR-A: scavenger receptor-A, STAT3: signal transducer and activator of transcription 3, TNF-*α*: tumor necrosis factor-*α*, and VCAM-1: vascular cell adhesion molecule-1.
